# Can New Technologies Make Us More Human? An Inquiry on VR Technologies in Social Cognition

**DOI:** 10.3389/fpsyg.2018.00705

**Published:** 2018-05-18

**Authors:** Daniel Żuromski, Adam Fedyniuk, Ewelina M. Marek

**Affiliations:** ^1^Department of Cognitive Science and Epistemology, Faculty of Humanities, Institute of Philosophy, Nicolaus Copernicus University in Torun, Torun, Poland; ^2^Kogni_LAB, Institute of Philosophy, Faculty of Humanities, Nicolaus Copernicus University in Torun, Torun, Poland

**Keywords:** social cognition, virtual reality, perspective taking, digital humanism, applied ontology, social ontology

## Introduction

There is a heavily ingrained image in contemporary culture, that new technologies can have a negative impact on human relations and bring us closer to dehumanization. For example the immersive worlds of games, virtual reality and social media, provide a surrogate of human relationships and interactions. Despite these dangers, we believe that new technologies can also make us more empathetic, altruistic and understanding toward each other. So we ask a question: can new, emerging technologies make us more human and become a factor in the process of humanization? In this article we present an idea that is a positive answer to this question, which could be called digital humanism. According to our thesis, new technologies extend and develop new tools of social cognition, especially when we consider the possibility to take perspectives of others that without the aid of said technologies would be impossible to comprehend. We can experience in first person, via VR technologies, how one suffers from various cognitive disorders and deficits like the ones associated with attention, autism, MCI (mild cognitive disorder), schizophrenia or color blindness. This refers to “digital” part of our thesis. The aforementioned experiences can influence, mainly through understanding and empathy, our moral actions and can overcome various biases like harmful stereotypes (Yee and Bailenson, 2006) or enhance altruistic attitudes in social cognition. At the same time it creates conditions for the formulation of new social relations (Bailenson, 2006; Bailenson et al., 2008). This refers to “humanism” in our thesis.

Even without using VR technology, perspective taking affects our behavior and attitude (Galinsky and Moskowitz, 2000). Generally speaking, taking perspective of another person (while interpreting him/her as intentional and equipped with a mind) is an essential human trait. It is a form of social cognition, which in turn is understood as:

General term used to describe different forms of cognition about, or actions in regard to, agents or groups of agents, their intentions, emotions, actions and so on, particularly in terms of their relation to other agents and the self (De Jaegher et al., 2010, p. 441).

The skills of perspective-taking also enable cultural learning, which allowed us, human species, development of new processes of sociogenesis and cumulative cultural evolution (Tomasello, 1999). In this approach, cultural practices and the culture itself are understood as abilities to create, accumulate and take perspectives of another person. For instance, through the works of Plato, we can take his historically accumulated perspective on various subjects. In contemporary culture, we also have other media that enable us to do the same. New technologies (like VR) help us develop new tools of social cognition that are essential to our species. Our aim is to present VR technologies as new tools of social cognition and to consider what path the modeling of social relations may take in the context of “extended mind” and procedurally modeled ontology-driven virtual reality.

## Empirical exemplification of digital humanism

To exemplify digital humanism, we refer to the studies of Ahn et al. (2013), Ahn et al. (2014) among other publications. Their study (Ahn et al., 2013) demonstrated how perspective taking while being immersed in VR environment affects dispositions attitudes and behavior. The mechanism that governs our affinity toward helping others is described as self-other merging which was used to explain the theoretical basis for the study (Ahn et al., 2013). A series of experiments was performed, that subjected participants to an environment that simulated perception of the world while suffering from colorblindness, others were instructed to only imagine such perspective. Experiment 1 was a comparison between the two groups in 24 h timeframe after. The results showed that participants with lower overall disposition toward helping others were affected more by the immersed experience. Experiment 2 showed that enhancing spatial immersion in VR also enhances longevity of the effects of the treatment and its impact. This was measured by a survey that took into account various parameters adapted from different psychological tests (the parameters were manipulation check, oneness, helping and attitude). Additionally participants wrote down their thoughts about the experience or the perspective they attempted to take. Experiment 3 was a demonstration of the effects of immersed VR experience in real world, and how sustainable they were. In that last part of the study, participants spent time helping colorblind people. The results showed a stronger impact of the VR experience compared to using solely traditional perspective taking.

To better understand the intricacies of perspective taking and perspective taking enhanced by VR experience, we need to take into account similarities and differences of those phenomena. Traditional perspective taking engages our imagination to assume others' point of view, to see through someone's eyes, so to speak. Virtual reality in this case gives us access to a “multilayer perceptual information simulated by digital device” (Ahn et al., 2013, p. 8) which allows us to see and hear as if we were experiencing someone else's point of view in the real world. Because of this multimodal aspect, we can call it an “embodied experience” (Ahn et al., 2013). Perspective taking has a substantial effect on social attitudes and cognition as well as learning (Tomasello et al., 1993). For instance, it has been found that it enhances empathy toward people from stigmatized social groups (Batson et al., 1997). Having said that, what is the difference between traditional perspective taking and embodied experience? Perspective taking requires significant cognitive resources (Roxβnagel, 2000) and also various factors that may motivate us to do it (Gehlbach et al., 2012). In case of embodied experience, seeing through someone's eyes is easier to achieve because of multimodal influence of VR.

Moreover, VR studies focus on the effect of embodying the physical traits of another person. During such investigations we can indicate the so-called “Proteus effect.” It is caused by changes in physical appearance of VR avatar that affect behavior of the person experiencing it. The results demonstrated that short amount of time in VR embodying a visual self-representation (i.e., an avatar) modified behavior of a person in the real world (Yee and Bailenson, 2006). For example, participants given attractive avatars in VR were more confident in interacting with strangers offline compared to those given unattractive avatars (Yee and Bailenson, 2009; Yee et al., 2009). It is speculated that the “Proteus Effect” is a different cognitive process than self-other merging mentioned above (Yee and Bailenson, 2009). Another way to influence our attitudes through embodied experiences is to diminish out-group and in-group racial bias by visualizing an avatar of different skin color than the participant (Maister et al., 2015; Hasler et al., 2017). There are also other studies that show the extent of the influence of VR environment that can affect real-world behavior (Peck et al., 2013; Ahn et al., 2014), as well as enhance prosocial behavior (Rosenberg et al., 2013; Shriram et al., 2017) and is considered to be used in health and education (De Oliveira et al., 2016). There is a need to investigate these processes further as well as the individual capacities, and socio-cultural practices they rely on.

## Theoretical aspects of digital humanism

Aside from the empirical studies, digital humanism is considered from a theoretical standpoint. From a purely theoretical standpoint, new VR technologies can be understood in the context of the “extended mind” conception of Clark and Chalmers (1998), according to which cognitive processes are not bound physically only by functioning of the brain, but are indeed extended into the agent's environment. In this view the distinction between the internal (within the person's cognitive system) and external (beyond the person's cognitive system) part of cognitive processes is arbitrary. In the context of digital humanism for the most part we take into consideration liberal interpretation by S. Gallagher (2013) who coined the term “social extended mind.” According to this account, “mental institutions” “not only accomplish certain cognitive processes but also are such that without them such cognitive processes would no longer exist” (Gallagher and Crisafi, 2009, p. 6). In our interpretation, VR technologies are a form of extended mind. In fact, they are “mental institutions” and they enable various cognitive processes, like aforementioned embodied experiences, which are in fact new tools of social cognition.

Taking this into account, knowledge about said mental institutions may be used as a model to further develop our understanding of social phenomena. So, how can we improve VR scenes for embodied experience? How can we be more precise in simulating intricacies of social dynamics within VR?

One of the possible ways of modeling and visualizing of these mental institutions with the use of new technologies may take into account social ontology, which can be understood as “a statement or understanding of the nature, character, or basic features, structures, or constituents of this. It is an explication or understanding of the basal “what there is” to social existence” (Woolgar and Schatzki, 2010). In our account, we consider social ontology in the sense of knowledge representation theories and its engineering apparatus (Davis et al., 1993). By creating social domain ontology, we can start to delve deeper into the analysis of its parameters and metrics. The data and parameters of social domain ontology can also be applied to VR technology. There are already methods of automated generation of virtual reality scenes representing domain defined in an ontology (SEMIC approach) (Flotynski, 2014). Additionally, SEMIC approach also can be understood as a type of procedural modeling method. By using a given set of instances of a domain ontology, the system automatically generates objects as contents of a VR scene. It uses provided data (properties, relations, hierarchy) to integrate it into set of objects to be used in an interactive environment. This provides an automated way to generate VR environments based on mental representations rather than aesthetic and design-driven standpoint. It may be a way create more accurate embodied experiences that would become a testing ground for social phenomena that incorporate mental institutions.

Combining virtual reality and knowledge representation allows for a unique way of designing and evaluating social mechanism models. The aforementioned automated generation of a fragment of a domain, (in this case, a social ontology) by using SEMIC, can become a scene for social interaction between two or more people connected to such virtual environment. There are two stages where mental representations of two or more subjects are combined—first, when two or more experts design domain ontology, their collective effort allows them to conceptualize said domain; second, for example, when two firefighters are immersed in a such SEMIC-generated VR scene of a burning building we can see how fast they can achieve mutual understanding by combining their representations to solve a problem. This can possibly give us new ways to view social interactions and also makes the transition of mental institutions from social domain to VR more seamless and specifically tailored to the user.

## Conclusions

In this Opinion we proposed a theoretical framework for conceptualization and systemic approach for further development of digital humanism. To be precise, we have described embodied experiences and VR technologies as a form of extended mind, more specifically socially extended mind and in turn as mental institutions that may be used as a model to further develop our understanding of social phenomena. We have given an example of how we can use domain ontologies to improve VR scenes for embodied experiences and how we can improve the simulation of details of social dynamics within collective embodied experiences.

We consider various forms of research in the future. More specifically, the analysis and creation of VR tools that enhance our social competencies and cognition. It is possible that VR technologies will become a common tool to diagnose, evaluate and refine these competencies. It would make VR not only a type of entertainment, but a formidable, widely-used scientific tool. For example, the use of VR might become equally important as questionnaires and surveys, especially in assessing employability skills. With this in mind, we may attempt to follow the development of VR technologies and how it will affect social cognition, or more generally, human mind.

## Author contributions

To emphasize the individual work put into this paper, Introduction, Theoretical Aspects of Digital Humanism, and Conclusions were written by DŻ and AF, while Empirical Exemplification of Digital Humanism was done by AF, DŻ, and EM.

### Conflict of interest statement

The authors declare that the research was conducted in the absence of any commercial or financial relationships that could be construed as a potential conflict of interest.
